# How ChatGPT transformed teachers: the role of basic psychological needs in enhancing digital competence

**DOI:** 10.3389/fpsyg.2024.1458551

**Published:** 2024-10-03

**Authors:** Jinshan Zhou, Lihan Shen, Weibang Chen

**Affiliations:** ^1^School of Education, Hainan Normal University, Haikou, Hainan, China; ^2^School of Education, Minzu University of China, Beijing, China; ^3^Department of Preschool Education, Hefei Preschool Education College, Hefei, Anhui, China

**Keywords:** ChatGPT, AI, teachers’ digital competence, self-determination theory, basic psychological needs

## Abstract

**Introduction:**

With the rapid development of ChatGPT, its application in the field of education has garnered widespread attention. This study aims to explore the impact of ChatGPT on teachers’ digital competence (TDC) and the mediating role of basic psychological needs satisfaction (BPNS).

**Methods:**

The study was conducted in China, collecting questionnaire data from 632 teachers through the QuestionStar platform. Structural equation modeling was employed using SmartPLS 4.0 to examine the effects of ChatGPT usage on TDC and its relationship with BPNS.

**Results:**

The findings indicate that ChatGPT has a significant effect on TDC, primarily through the fulfillment of competence and relatedness needs, while the impact of autonomy on TDC was not significant.

**Discussion:**

The results indicate that ChatGPT can enhance TDC and improve intrinsic motivation by satisfying their basic psychological needs. It is recommended that the design of educational tools consider teachers’ psychological needs to promote their professional development and well-being. This provides practical guidance for educational institutions, emphasizing the importance of teachers in the digital transformation process.

## 1 Introduction

The advent of the ChatGPT in November 2022 garners global attention. GenAI, with its ability to generate complex and realistic content resembling human creativity, has become a valuable tool across various industries such as education, entertainment, and product design, with a profound and lasting impact on these fields. The utilization of ChatGPT in education presents prospects for surmounting geographical constraints imposed by national and international boundaries, as educational resources are now readily available on the internet and on the global Web (Chen et al., 2020). This platform offers educators a wide range of resources, including detailed descriptions of innovative teaching methods, cutting-edge technologies, and up-to-date materials. These resources help teachers stay informed about the latest advancements in education (Kasneci et al., 2023).

Specifically, teachers can utilize ChatGPT to achieve personalized learning, create or modify instructional materials, draft emails in response to student inquiries, and engage in interdisciplinary teaching (Chiu, 2024). Through the analysis of data regarding effective teaching methods and student academic achievements, ChatGPT can provide individualized suggestions to assist teachers in enhancing their instructional techniques (Ray, 2023). ChatGPT functions as a virtual tutor, effectively supporting self-directed learning among adult learners in asynchronous online environments by assisting with goal setting, resource discovery, personalized learning plans, and performance monitoring (Lin, 2024). Teachers using ChatGPT can utilize it both for learning and as an aid in the teaching process. The resources provided by ChatGPT undoubtedly promote teachers’ professional development and enhance their knowledge and skills.

Currently, there are two notable developments in the field of education. First, educational systems across the globe are integrating digital competence into their instructional materials and evaluations. Second, teachers are actively encouraged to incorporate technology into their teaching methods, either as tools to enhance learning or as a means for informal evaluation. These trends highlight the growing importance of teachers’ digital competence (TDC) in the GenAI era (Scherer et al., 2019). Moreover, while much research has focused on integrating AI into classrooms (Karumbaiah et al., 2023; Chen et al., 2023; Jeon, 2024), teachers often lack sufficient training in digital technology (Fernández-Batanero et al., 2022, Gudmundsdottir and Hatlevik, 2018, Napal Fraile et al., 2018), resulting in underdeveloped TDC. Furthermore, digital education introduces challenges such as changes in the teacher’s role, reduced face-to-face interaction, and increased workloads, all of which require improvements in TDC (Babushko et al., 2022). Exposure to new digital tools can motivate teachers to incorporate various technologies into the classroom, thus enhancing their digital competence (Timotheou et al., 2023). ChatGPT, as a leading example of GenAI, offers rich resources, ease of use, and convenience, helping to address the challenges teachers face in developing TDC. Most previous studies have explored the impact of ChatGPT on students, with limited attention on teachers. Notably, a gap remains in research regarding TDC.

In addition, this study examined the influence of satisfaction of basic psychological needs as described in Self-Determination Theory (SDT). SDT focuses on people’s intrinsic motivational tendencies to learn and grow and how to support them (Ryan and Deci, 2020). Satisfaction of basic psychological needs is essential for psychological health, growth, autonomous motivation, optimal functioning, and self-actualization (Deci and Ryan, 2008). This is especially true for teachers, as they face many crises that undermine their motivation, such as school stress, classroom overload, and disruptive student behavior (Fernet et al., 2012, Kaplan and Madjar, 2017). If teachers effectively satisfy the three basic psychological needs—autonomy, competence, and relatedness—through their use of ChatGPT, it may foster continuous progress in their digital competence, which is necessary for the development of TDC. This study utilized ChatGPT usage by teachers as the independent variable, basic psychological needs satisfaction as the mediator, and TDC as the dependent variable. SmartPLS 4.0 was used to construct structural equation models to investigate the effect of ChatGPT on TDC.

## 2 Literature review and hypotheses

### 2.1 Teacher digital competence

With the growing recognition of the importance of integrating technology into education (Yilmaz et al., 2024; Yılmaz and Yılmaz, 2023), notable frameworks for assessing teachers’ digital competence include SAMR (Puentedura, 2012), TPACK (Mishra and Koehler, 2006), UNESCO (United Nations Educational, Scientific and Cultural Organization, 2011), ISTE (Crompton, 2017), and DigCompEdu (Redecker, 2017). Among these frameworks, DigCompEdu, organized into six dimensions, has been validated as a reliable and effective tool for measuring teachers’ digital competence (Ghomi and Redecker, 2019). Cabero-Almenara et al. (2020) further emphasized that the DigCompEdu framework effectively assesses TDC. The DigCompEdu framework defines digital competence as the ability to use technology securely, critically, and innovatively to achieve educational goals (Redecker, 2017). This framework applies to educators at all levels, from kindergarten to university instructors. It functions at three levels—micro, meso, and macro—supporting and guiding teachers’ practices and ongoing professional development (Skantz-Åberg et al., 2022). Therefore, this study employs the DigCompEdu framework to evaluate the TDC.

Prior research has shown that simplicity, trust in the use of digital technology, and acceptance of new technology are all important indicators of digital technology proficiency (Lucas et al., 2021; Gil-Flores et al., 2017). Teachers’ attitudes toward the digital skills required for technology integration in teaching are closely related to their perceptions of the technology’s usefulness and their plans for its adoption (Antonietti et al., 2022). Galindo-Domínguez et al. (2024) reported a positive correlation between TDC and teachers’ attitudes toward AI in education, regardless of personal variables such as educational stage, gender, age, years of experience, or field of knowledge. However, opposing views suggest that optimism about technology does not automatically lead to behavioral change (Belland, 2009; Instefjord and Munthe, 2017). In other words, positive attitudes toward AI technology do not necessarily enhance teachers’ digital competence. Based on the above research, we propose Hypothesis 1.

Hypothesis 1: ChatGPT positively influences TDC.

### 2.2 Self-determination theory and basic psychological needs satisfaction

Developed by Deci and Ryan in the 1980s, SDT provides a comprehensive perspective on motivation and behavior (Deci and Ryan, 2013). At the core of SDT is basic psychological needs satisfaction (BPNS), which posits that motivation is driven by three fundamental needs: autonomy, competence, and relatedness (Ryan and Deci, 2020). Autonomy reflects an individual’s control over their actions, supported by personal interests and values, but it can be undermined by external controls like rewards or punishments. Competence, the sense of mastery and potential for growth, thrives in environments that provide optimal challenges, positive feedback, and developmental opportunities. Finally, relatedness involves a sense of belonging and connection, facilitated by respect and caring. The obstruction of any of these three basic needs is considered detrimental to motivation and health. SDT analyzes educational environments by examining how they satisfy or impede these basic needs; therefore, our study explores how ChatGPT meets these three basic psychological needs.

Current research has explored the impact of perceived support on teachers’ BPNS, including school support (Chiu et al., 2024), principal’s learning support (Collie et al., 2016; Klaeijsen et al., 2018; Lee et al., 2020), teaching, and the social presence of teachers (Turk et al., 2022). Ryan and Deci (2020) highlight a growing research trend in SDT focused on the opportunities and challenges of new technologies in education. Teachers’ motivation to use technology as a learning tool is expected to become a more prominent area of study. However, there is still limited research on the impact of ChatGPT on teachers’ BPNS.

ChatGPT acts as a pedagogical assistant, helping teachers overcome challenges in their practice. It saves time, allows teachers to work at their own pace, and promotes autonomy and independence in lesson planning and instruction. Additionally, ChatGPT can assist teachers in analyzing data on students’ learning preferences, strengths, and weaknesses to design personalized learning experiences (Baidoo-Anu and Ansah, 2023). ChatGPT has demonstrated significant advantages and potential, particularly in assisting programming learning (Yilmaz and Yilmaz, 2023a,b). ChatGPT aids teachers in optimizing their instructional design and enhancing the academic performance of their classes by providing learning materials and tailored advice. Efficient task completion likely fosters a sense of competence and mastery in teachers. In terms of relatedness, AI can assist teachers in identifying student emotions like engagement, boredom, and frustration. This helps tailor instructional design and content to strengthen teacher–student relationships, as these emotions may be easily overlooked by teachers (Standen et al., 2020). ChatGPT also reduces isolation among colleagues by offering opportunities for teachers to share tips and exchange experiences. Both Shen and Cui (2024) and Chiu (2022) employed quantitative methods, demonstrating that technical support can enhance BPNS. Lu et al. (2023) found that AI robots significantly boost learners’ intrinsic motivation. Compared to traditional methods, AI-assisted learning more effectively stimulates teachers’ intrinsic motivation and improves learning outcomes (Fidan and Gencel, 2022). Furthermore, multiple studies have highlight ChatGPT’s significant potential to stimulate intrinsic motivation, particularly in learning (Ali et al., 2023; Zhou and Li, 2023; Wu et al., 2024). On the basis of the above research, the following hypothesis was formulated:

Hypothesis 2: ChatGPT positively influences teachers’ BPNS.

Additionally, BPNS significantly influences TDC by enhancing teachers’ motivation to use digital resources (Salikhova et al., 2020). A previous study indicated that teachers’ motivation to continue using digital technology is driven by perceived autonomy, competence, and relatedness (Sørebø et al., 2009). Consequently, BPNS has frequently been used to study the enhancement of digital competence and literacy. For example, Chiu et al. (2022) found that BPNS mediate the relationship between perceived technological learning assistance and digital literacy in students. In this context, autonomy and competence may be more important than relatedness. A recent study by Chiu et al. (2024) revealed that BPNS positively impacts TDC. Thus, Hypothesis 2 is formulated:

Hypothesis 3: BPNS positively influences TDC.

## 3 Methods

### 3.1 Samples

The study was conducted in China, using data collected through an anonymous, voluntary questionnaire distributed via the QuestionStar platform. The participants were teachers from 20 cities across China, including Beijing, Shanghai, and Guangdong, among others. This broad geographic distribution increased the sample diversity.

The sample size was determined based on recommendations suggesting it should be approximately 10–20 times the number of observed variables in the model (Schreiber et al., 2006; Kline, 2023). With 43 observed variables, the final questionnaire required at least 430 completed responses. However, to account for potential incomplete responses, the target was rounded up to 500. A total of 661 questionnaires were initially collected. After the exclusion of invalid questionnaires, such as those with excessively short response times or uniform answers, 632 valid responses remained, consisting of 442 females and 190 males. The age distribution was relatively balanced: 97 participants were aged 18–25, 155 were aged 26–30, 132 were aged 31–40, 117 were aged 41–50, and 131 were over 50. Teaching experience varied: 265 had 0–5 years, 78 had 6–10 years, 49 had 11–15 years, and 240 had over 16 years of experience. Educational backgrounds varied: 43 held an associate degree or below, 496 held a bachelor’s degree, 75 held a master’s degree, 15 held a doctoral degree, and 3 chose not to disclose their education level. Additionally, the teaching locations included 139 participants from rural schools, 186 from township schools, 297 from urban schools, and 10 who chose not to disclose their location.

### 3.2 Research instruments

The research instrument included a background section (Part 1) and a questionnaire for teachers (Parts 2–4). Part 1 collected information on each teacher’s gender, age, teaching experience, educational background, and teaching location.

#### 3.2.1 Basic psychological needs

Part 2 included 13 items related to teachers’ BPNS, adapted from Chen et al. (2015). This scale measures three dimensions: autonomy, competence, and relatedness. The autonomy dimension includes four items, such as “I feel I have choice and freedom in my daily tasks.” The competence dimension comprises four items, such as “I feel capable of achieving my work objectives.” The relatedness dimension includes five items, such as “I feel warmth in my interactions with others.”

#### 3.2.2 Teacher digital competence

Part 3 consisted of 15 items related to TDC, adapted from the European Framework for the Digital Competence of Educators (Redecker, 2017). Example questions include “I can create customized digital materials that cater to specific teaching and learning needs” and “I can use digital technologies to provide tailored learning solutions.” The TDC scale was chosen for its widespread recognition in assessing educators’ ability to effectively integrate digital tools into their teaching, which is crucial in the digital transformation of education.

#### 3.2.3 Use of ChatGPT

Finally, Part 4 included 15 items related to ChatGPT, adapted from Yilmaz et al. (2023). This scale measures four dimensions: performance expectancy, effort expectancy, facilitating conditions, and social influence. Example questions include “ChatGPT improves my efficiency in completing tasks” and “If I encounter a problem with ChatGPT, I can find a solution.”

#### 3.2.4 Expert evaluation

After the questionnaire was developed, it was evaluated by a panel of experts. Based on the evaluation, the questionnaire items were adjusted for clarity. The final questionnaire employed a seven-point Likert scale to measure items in Parts 2–4 (Pimdee et al., 2023; Sukkamart et al., 2023).

### 3.3 Data analysis

SPSS29 was used for the correlation analysis of variables in this study. The data were further analyzed using SmartPLS4.0, applying the Partial Least Squares Structural Equation Modeling (PLS-SEM) technique. PLS-SEM was selected because it is particularly suitable for handling complex models with relatively small sample sizes (Hair et al., 2019).

## 4 Results

### 4.1 Descriptive statistics and correlation analysis

SPSS 29 was used to conduct statistical analyses on all data. Descriptive statistics highlighted general trends, while correlation analysis examined the strength and direction of relationships between the variables. Table 1 presents the descriptive statistics and bivariate correlations for the five variables. The correlations between autonomy and ChatGPT were weak, whereas correlations among the other variables ranged from moderate to strong. The average score for each variable indicated the level of consensus among participants regarding the survey statements. Overall, teachers exhibited a high level of TDC, though their autonomy was relatively low.

**Table 1:** Descriptive statistics and correlation analysis.

Variable	1	2	3	4	5
1.AU	–				
2.CO	0.576**	–			
3.RE	0.577**	0.577**	–		
4.TDC	0.443**	0.589**	0.513**	–	
5.ChatGPT	0.39**	0.488**	0.487**	0.675**	–
Mean	5.323	5.798	5.637	5.829	5.457
SD	1.239	0.938	0.911	0.767	1.008
Skewness	−0.788	−1.143	−0.623	−0.616	−0.532
Kurtosis	0.327	2.114	0.185	0.261	−0.079

**Correlation is significant at the 0.01 level (two-tailed).

AU, Autonomy; CO, Competence; RE, Relatedness; TDC, Teachers’ digital competence.

### 4.2 Measurement model evaluation

Table 2 presents the Cronbach’s Alpha, Combined Reliability, AVE, and Heterotrait-Monotrait Ratio for each latent variable. The Cronbach’s Alpha and Combined Reliability values for each latent variable were greater than the critical value of 0.7, indicating good reliability of the measurement model. The AVE values were all above 0.5, showing acceptable convergent validity (Hair et al., 2019). The HTMT values were below 0.85, indicating sufficient differentiation between the variables (Henseler et al., 2015). In summary, the model was measured and confirmed to have good reliability and validity.

**Table 2:** Reliability, convergent validity, and discriminant validity of the model.

	AU	CO	ChatGPT	RE	TDC
AU					
CO	0.647				
ChatGPT	0.426	0.520			
RE	0.664	0.654	0.539		
TDC	0.483	0.639	0.711	0.576	
Cronbach’s alpha	0.876	0.903	0.961	0.854	0.948
Composite reliability (rho_a)	0.877	0.906	0.962	0.855	0.948
Composite reliability (rho_c)	0.915	0.932	0.965	0.896	0.953
AVE	0.730	0.774	0.649	0.633	0.578

### 4.3 Evaluation of the structural model

**Figure 1:**
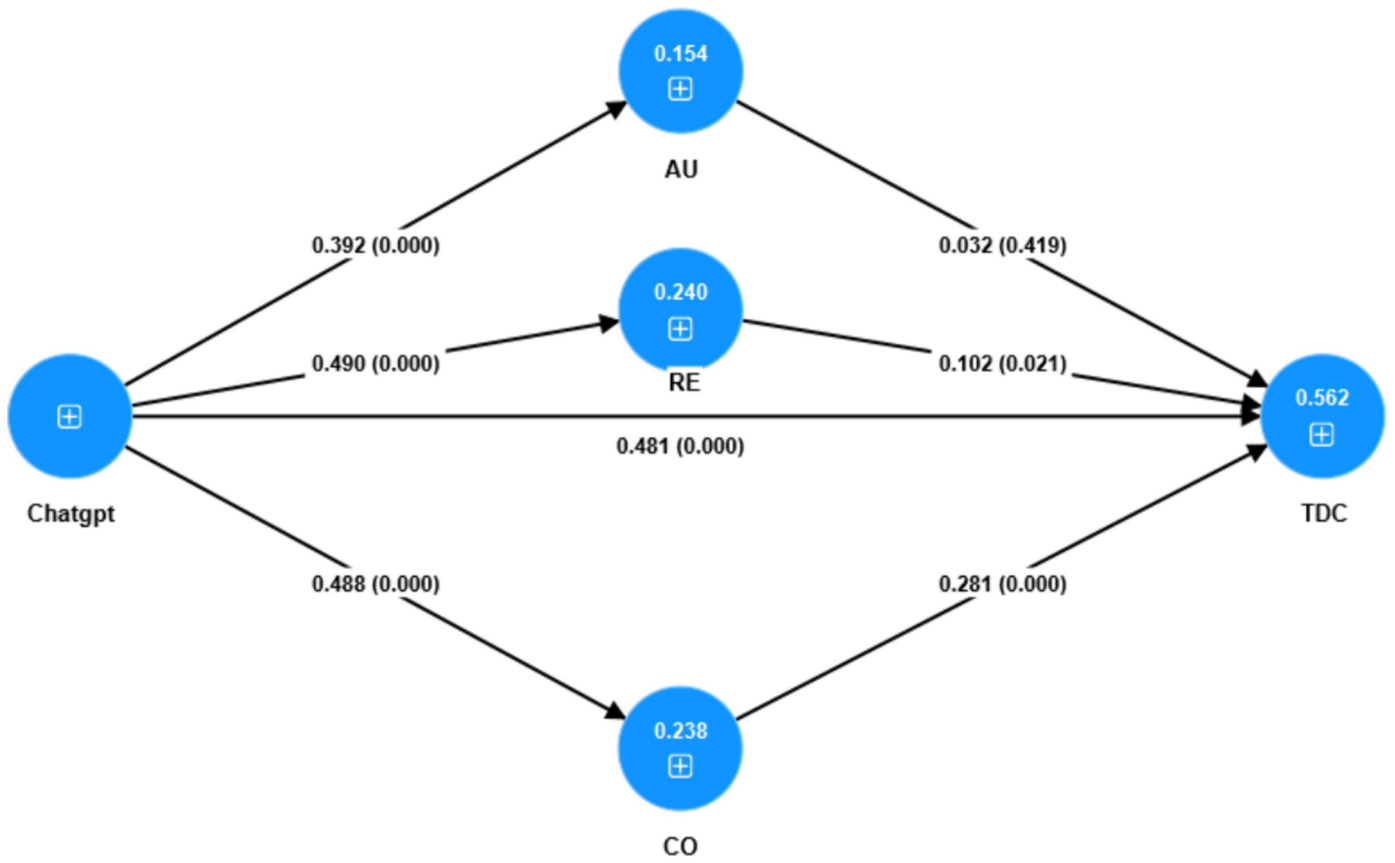
Path coefficients, *p* values, and *R*^2^ values of the model.

Partial Least Squares Structural Equation Modeling was utilized in this study on the collected questionnaire data. Hypothesis testing was conducted using SmartPLS4.0. Random sampling was carried out using the Bootstrapping technique, with a predetermined sample size of 5,000. The results are shown in Figure 1. ChatGPT had a significant effect on TDC and contributed to TDC through the satisfaction of competence and relatedness among BPNS. However, the relationship between autonomy and TDC was non-significant. The coefficients of determination (*R*^2^) for autonomy, competence, relatedness, and TDC were 0.154, 0.238, 0.24, and 0.562, respectively. The total explanatory power of the research model was 0.562, indicating that the model had strong predictive ability for TDC.

### 4.4 Hypotheses testing results

Table 3 and Figure 1 present the hypothesis testing results. ChatGPT had a positive and significant impact on TDC, establishing Hypothesis 1. The effects of ChatGPT on autonomy (*β* = 0.392, *p* < 0.001), relatedness (β = 0.490, *p* < 0.001), and competence (β = 0.488, *p* < 0.001) were all positive and significant, confirming Hypothesis 2. Three mediators—autonomy, relatedness, and competence in BPNS—were set up in this study. The impacts of relatedness (β = 0.102, *p* < 0.05) and competence (β = 0.281, *p* < 0.001) on TDC were positive and significant, but the impact of autonomy on TDC was not significant (β = 0.032, *p* > 0.05). Both relatedness and competence were significant mediators, but autonomy was not. Hypothesis 3 could only be partially confirmed.

**Table 3:** Decomposition of direct (DE), indirect (IE), and total (TE) effects of the SEM.

Path	Path coefficient	95% confidence intervals	T statistics	*p* values	Significance
Direct effects					
AU - > TDC	0.032	[−0.046,0.112]	0.809	0.419	No
CO - > TDC	0.281	[0.193,0.374]	6.035	<0.001	Yes
ChatGPT - > AU	0.392	[0.316,0.472]	9.836	<0.001	Yes
ChatGPT - > CO	0.488	[0.410,0.569]	11.975	<0.001	Yes
ChatGPT - > RE	0.490	[0.423,0.557]	14.337	<0.001	Yes
ChatGPT - > TDC	0.481	[0.403,0.559]	12.106	<0.001	Yes
RE - > TDC	0.102	[0.015,0.186]	2.316	<0.05	Yes
Indirect effects					
ChatGPT - > AU - > TDC	0.013	[−0.018,0.045]	0.795	0.427	No
ChatGPT - > CO - > TDC	0.137	[0.091,0.193]	5.276	<0.001	Yes
ChatGPT - > RE - > TDC	0.05	[0.007,0.091]	2.337	<0.05	Yes
Total indirect effects					
ChatGPT - > TDC	0.200	[0.150,0.255]	7.550	<0.001	Yes

## 5 Discussion

### 5.1 Research implications

This study investigated the impact of ChatGPT on teachers’ TDC while also examining the influence of ChatGPT on teachers’ BPNS and the subsequent effect of BPNS on TDC. The results are summarized below.

The first hypothesis examined the effect of ChatGPT on TDC. Although direct studies on ChatGPT’s impact on TDC are limited, our findings indicate that ChatGPT has significant potential to enhance TDC. This aligns with previous research emphasizing the role of attitudes toward new technologies in shaping digital skills. For example, Lucas et al. (2021) found that ease of use, teachers’ confidence in digital technologies, and openness to new technologies predict TDC. Galindo-Domínguez et al. (2024) identified a positive correlation between TDC and teachers’ attitudes toward AI in education, independent of factors such as educational stage, gender, age, years of experience, or field of knowledge. Combining the analysis of previous studies with our findings, we conclude that ChatGPT can enhance TDC. Beyond recognizing ChatGPT’s positive impact on TDC, we also propose that it serves as a tool that fosters both adaptability and innovation in teachers’ use of digital tools. By making AI-powered educational resources more accessible, ChatGPT helps teachers build confidence and familiarity with emerging technologies, ultimately contributing to the development of TDC. In the face of continuous technological advancement, adopting GenAI technologies like ChatGPT is crucial for helping teachers navigate the rapidly evolving digital landscape.

The second hypothesis examined the relationship between ChatGPT and teachers’ BPNS. This result suggests that the use of ChatGPT helps to fulfill teachers’ basic psychological needs for autonomy, competence, and relatedness, thus increasing their intrinsic motivation. This result is consistent with Al-Rahmi et al. (2020), who found that the use of ICT can promote BPNS. Studies by Shen and Cui (2024) and Chiu (2022) also demonstrated that technical support facilitates BPNS. Lu et al. (2023) indicated that AI robots significantly enhance learners’ intrinsic motivation. Thus, it can be concluded that ChatGPT positively influences teachers’ BPNS. This finding has important implications for the design of AI-powered educational tools. Specifically, tools like ChatGPT should be designed to not only enhance TDC but also support teachers’ mental health and overall development. Technology design should ensure that these tools are harmoniously integrated with teachers’ inner selves, promoting both professional growth and emotional well-being.

The third hypothesis tested whether BPNS promotes TDC, and the results were only partially supportive. The fulfillment of different needs affects the TDC differently. Competence and relatedness have significant positive impacts on TDC, with path coefficients of 0.281 (*p* < 0.001) for competence and 0.102 (*p* < 0.05) for relatedness. This suggests that an increased sense of competence and relatedness, as perceived by teachers after using ChatGPT, further enhances their digital competence. These findings align with Bergdahl et al. (2023), who noted that competence and relatedness are positively correlated with AI positivity. However, the path coefficient of autonomy on TDC was only 0.032 (*p* = 0.419), indicating that the direct effect of autonomy on TDC was not significant, which contrasts with our predictions. One possible explanation is that some teachers have not yet mastered the effective use of ChatGPT. Although some teachers adopted a positive attitude toward ChatGPT, they had not yet mastered how to effectively prompt it to generate desired responses. This lack of mastery, potentially due to insufficient training and practical experience, contributed to the failure to improve TDC. Moorhouse (2024) noted that new teachers often find that AI-generated content does not meet their expectations. For instance, one interviewed teacher stated, “whatever they generate is different from what I want.” If teachers lack effective usage strategies or are aware of the limitations of ChatGPT, they may not feel digitally empowered when they use these tools. One possible explanation is that teachers with high levels of autonomy have significant responsibility for their actions and their students. Some teachers are concerned about potential bias and privacy violations related to AI technologies, which affects their trust in and willingness to use these tools (Omrani et al., 2022). Haseski (2019) noted that there could be hidden hazards to the well-being of teachers and students associated with AIEd, which may erode teachers’ trust and reliance on AI tools in the classroom. This may also result in a non-significant relationship between autonomy and TDC, as the limitations of the tools can hinder teachers’ use and exploration of them. This underscores the need to address teachers’ concerns about AI tools and to provide targeted professional development, ensuring they feel confident and autonomous in using these technologies. Furthermore, effective use of technological tools often requires adequate support and training. Without sufficient training, even if teachers’ autonomy is fulfilled, they may not find effective ways to improve TDC due to a lack of appropriate skills. Therefore, adequate teacher training is essential.

### 5.2 Practical applications

Based on the results, we propose the following recommendations for teachers and educational institutions:

For teachers, adopting an open and inclusive attitude toward emerging technologies such as ChatGPT is essential. Actively experimenting with and exploring these tools not only enhances their technological skills but also enriches the learning experiences of students. Teachers should embrace technological challenges and continuously apply and adapt these tools in their teaching practices to improve their digital competence. Only with strong digital competence can teachers effectively implement AI-driven teaching tools to support teaching and learning in the future. Furthermore, teachers should develop tailored professional development initiatives to address the specific challenges and issues related to implementing artificial intelligence in educational settings (Kohnke et al., 2023).

According to Skantz-Åberg et al. (2022), the collective efforts of individual teachers alone are insufficient for achieving digital transformation. School leadership must actively support and advance the process of digitization by formulating clear strategies and plans for digital technology, providing teachers with essential digital resources and training, and ensuring the consistent and equitable use of technology in teaching and learning. Leadership support is crucial for enhancing teachers’ digital competence. When considering teacher training, the following points should be noted: Online training is a primary method for improving the digital skills of currently employed teachers (Yang et al., 2023). It offers flexibility by allowing teachers to study outside of work hours and adjusting to their individual progress. This approach ensures that the training meets teachers’ specific learning needs. Training content must be regularly updated to keep pace with technological advancements. For example, ChatGPT has rapidly evolved from ChatGPT-3 to the current ChatGPT-4. The training content should reflect the rate of technological growth. In-service teacher training strategies should focus on teachers’ sense of relatedness and competence. Organizing group discussions and exchange sessions can enhance interaction and cooperation among teachers, increasing their participation and motivation to learn. Additionally, tasks should be designed to avoid excessive complexity to foster teachers’ sense of competence and self-confidence.

### 5.3 Limitations and future research

While this study demonstrated the positive impact of ChatGPT on enhancing TDC, several limitations must be acknowledged. First, although participants were drawn from 20 different cities across China, the study is confined to the Chinese educational context and does not explore other countries. Cultural background factors significantly influence the adoption and integration of educational technologies into teaching practices. The results may reflect attitudes toward technology, education, and innovation that are specific to China, where rapid digitalization and strong government support for educational technology have created a particularly conducive environment for the adoption of AI tools. Consequently, the applicability of these findings to educational settings in other countries, especially those with different cultural and technological environments, may be limited.

Second, the study focused primarily on teachers at the elementary, middle, and high school levels, leaving the generalizability of the findings to preschool and higher education teachers uncertain. This limitation suggests that the study’s findings may not be applicable across all levels of education.

Third, this study did not include an intervention, and the lack of control over all external variables that could affect TDC, such as the level of technological support in schools, teachers’ personal interests, and technological backgrounds, represents a significant limitation. These factors could have influenced the results, and future research should aim to control as many external variables as possible to increase the accuracy and reliability of the findings.

Moreover, this study employed a cross-sectional design with a short research period, which limits the ability to observe the long-term effects of ChatGPT on TDC. This design restricts the depth of insight into the enduring impact of ChatGPT on BPNS and TDC. Future research should consider using a longitudinal design to assess the long-term impact of ChatGPT on these variables more comprehensively.

Finally, while the study utilized established instruments for measuring the constructs, there is room for reflection on the appropriateness and sensitivity of these tools within this specific context. The choice of instruments, along with the statistical outcomes derived from them, could introduce biases or limitations that should be acknowledged. A more reflective approach in future research regarding instrument selection and the interpretation of statistical results would enhance the robustness of findings. Notably, the DigCompEdu framework used in this study, though a widely recognized tool, lacks strong theoretical grounding, which may undermine its robustness and applicability. Future research should consider addressing this theoretical gap and carefully evaluate the choice of instruments and the interpretation of statistical results to enhance the robustness and validity of findings.

## 6 Conclusion

This study investigates the effects of ChatGPT on teachers’ digital competence and the satisfaction of their basic psychological needs. The findings demonstrate that ChatGPT significantly enhances teachers’ digital competence and promotes the satisfaction of their basic psychological needs, with competence and relatedness serving as mediators. However, autonomy does not mediate this relationship, as its effect on teachers’ digital competence is non-significant.

The study contributes to the current body of research by addressing the gap in understanding how AI tools like ChatGPT can impact both teachers’ teaching effectiveness and their psychological well-being. Our results indicate that ChatGPT has a favorable impact on these aspects, aligning with and expanding upon existing research. These findings underscore the importance of integrating AI tools into educational practices to enhance both instructional effectiveness and teacher well-being.

Furthermore, the study provides actionable recommendations for improving digital competence, highlighting the need for both individual teacher efforts and institutional support. These insights offer valuable guidance for educational policy and practice.

In conclusion, while the study offers significant contributions, it also recognizes its limitations, particularly in the scope of data and the generalizability of findings. Future research is encouraged to further explore the impact of AI tools like ChatGPT on different educational settings and to examine other potential mediating factors. This will help build a more comprehensive understanding of how AI can support the evolving needs of teachers and educational institutions.

## Data Availability

The raw data supporting the conclusions of this article will be made available by the authors, without undue reservation.
